# Anion-Enriched Interfacial Chemistry Enabled by Effective Ion Transport Channels for Stable Lithium Metal Batteries

**DOI:** 10.3390/ma18112415

**Published:** 2025-05-22

**Authors:** Yi Li, Hongwei Huang, Haojun Liu, Dedong Shan, Xuezhong He, Lingkai Kong, Jing Wang, Qian Li, Jian Yang

**Affiliations:** 1 College of Materials Science and Engineering, Jiangsu Collaborative Innovation Center for Advanced Inorganic Function Composites, Nanjing Tech University, Nanjing 211816, China; liyi19991225@163.com (Y.L.); hhw2317452719@163.com (H.H.);; 2 Guangdong Provincial Key Laboratory of Materials and Technologies for Energy Conversion, Department of Chemical Engineering, Guangdong Technion-Israel Institute of Technology, Shantou 515063, China; xuezhong.he@gtiit.edu.cn; 3 Longdu Laboratory for New Chemical Materials, Puyang 457000, China

**Keywords:** anion-rich interphase, solvation structures, lithium metal batteries, anion-derived SEIs

## Abstract

The formation of unstable solid electrolyte interphases (SEIs) on the surface of lithium metal anodes poses a significant barrier to the commercialization of lithium metal batteries (LMBs). Rational modulation of solvation structures within the electrolytes emerged as one of the most effective strategies to enhance interfacial stability in LMBs; however, this approach often compromises the structural stability of the bulk electrolyte. Herein, we present an innovative method that improves interface stability without adversely affecting the bulk electrolyte’s structural stability. By employing ZSM molecular sieves as efficient ion channels on the lithium metal anode surface—termed ZSM electrolytes—a more aggregated solvation structure is induced at the lithium metal interface, resulting in an anion-rich interphase. This anion-enriched environment promotes the formation of an SEI derived from anions, thereby enhancing the stability of the lithium metal interface. Consequently, Li||Cu cells utilizing the ZSM electrolyte achieve an average coulombic efficiency (CE) of 98.76% over 700 h. Moreover, LiFePO_4_||Li batteries exhibit stable cycling performance exceeding 900 cycles at a current density of 1 C. This design strategy offers robust support for effective interfacial regulation in lithium metal batteries.

## 1. Introduction

With the rapid advancement of portable electronic devices and electric vehicle technologies, there is an urgent demand for high-energy-density lithium-ion batteries (LIBs) [1,2]. Current commercial LIBs, particularly those utilizing graphite anodes, nearly reached their theoretical energy density limits. Transitioning to lithium metal anodes, which boast a significantly higher theoretical capacity (3860 mAh g^−1^) and lower electrochemical potential (−3.04 V vs. standard hydrogen electrode), could substantially elevate battery energy densities [3,4,5,6,7]. However, the development of unstable solid–electrolyte interphases (SEIs) on the lithium metal surface severely impacts the coulombic efficiency (CE) of lithium plating/stripping, potentially leading to uncontrolled dendrite growth [8,9,10,11,12,13,14]. Therefore, rationally regulating SEI structures/properties is critical for stabilizing lithium metal batteries.

Over the past few decades, numerous strategies have been proposed to improve the interfacial stability of lithium metal anodes, including electrolyte structure optimization [15], electrode surface modification [16], and interfacial property regulation [17]. Among these approaches, modulating the electrolyte structure to stabilize the SEI has proven particularly effective because it directly influences SEI composition and structure [18,19,20,21,22]. High-concentration electrolytes (HCEs) represent one such method, Fan et al. successfully inhibited the growth of lithium dendrites by increasing the concentration of LiFSI to 10 M in carbonate electrolytes; Xu et al. designed a ‘water-in-salt’ electrolyte to improve lithium-ion battery performance [23,24,25,26]. The solvation structures evolve from solvent-separated ion pairs (SSIPs) to contact ion pairs (CIPs), fostering more stable anion-derived SEI formation [27,28,29,30,31]. However, HCEs present challenges such as high cost due to expensive lithium salts, increased viscosity, and poor wettability, limiting their practical applications [32,33]. To mitigate these issues, local high concentration electrolytes (LHCEs) have been developed by incorporating inert components into HCE. Lin et al. designed a THF-based localized high-concentration electrolyte to successfully improve the adaptability of lithium metal batteries in low-temperature environments [34,35,36]. Yet, adding inert solvents can reduce the overall energy density of lithium batteries. Recently, weak solvating electrolytes (WSEs) emerged; using solvents with limited lithium salt solubility that promote aggregated solvation structures conducive to anion-derived SEI formation, Li et al. Developed a DPE-based low-polarity electrolyte, which successfully formed a more robust CEI at the anode interface [37,38,39,40,41]. Despite this advantage, insufficient lithium salt dissolution in WSEs can impede Li^+^ transport within the electrolyte. The essence of these strategies lies in forcing anions into the solvation sheath to encourage anion-derived SEI formation, thereby enhancing interface stability. However, excessive incorporation of anions into solvation structures in bulk electrolytes often introduces drawbacks, such as high viscosity, low conductivity, or instability. Consequently, there is an urgent need for methods that enhance the interfacial stability of lithium metal anodes while preserving the overall stability of the bulk electrolytes.

**Figure 1:**
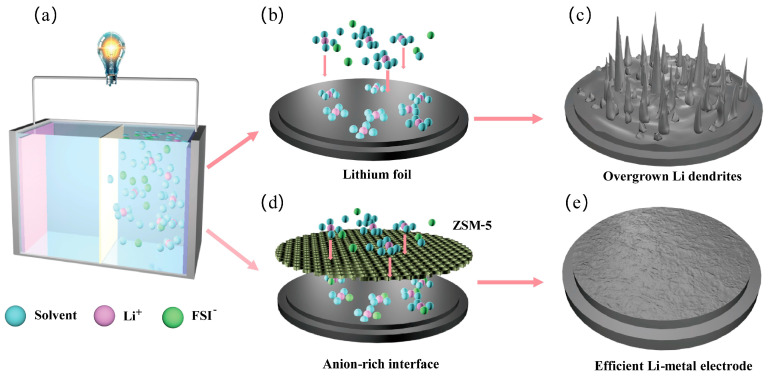
Schematic diagram of the lithium dendrite inhibition mechanism in the ZSM electrolytes. (**a**) Schematic diagram of the Li-metal battery. (**b**) Schematic of lithium metal anode with DME electrolytes. (**c**) The original interface results in Li dendrites formation. (**d**) Schematic of lithium metal anode with ZSM electrolytes. (**e**) The anion-rich interface promoting uniform lithium deposition on the Li-metal surface enhances the efficiency of the Li-metal electrode.

In this work, we employed ZSM-5 molecular sieves as effective ion-selective channels at the lithium-metal anode interface without changing the composition of the native electrolytes (hereafter referred to as ZSM electrolytes) [42,43,44]. The uniform pore structure within the molecular sieve efficiently expels a portion of the solvent from the solvation shell, creating an anion-rich interphase that facilitates the formation of anion-derived SEIs (Figure 1). The enhanced presence of LiF components in these anion-derived SEIs significantly improves the stability of the lithium metal interface. Consequently, Li||Cu cells utilizing ZSM electrolytes achieve an average CE of 98.76% over 700 h. Moreover, LiFePO_4_||Li batteries exhibit stable cycling performance for up to 900 cycles at a current density of 1 C.

## 2. Experimental

### 2.1. Preparation of ZSM-5@Celgard Separator

(1) Activation/Water Removal Process of Zeolite Powder

The ZSM-5 molecular sieves ((SiO_2_)x(Al_2_O_3_)y) were sourced from Shanghai MackLin Biochemical Co., Ltd. (Shanghai, China), while the Celgard 2500 separators were obtained from Suzhou Duoduo Chemical Technology Co., Ltd. (Suzhou, China). To prepare the ZSM-5 molecular sieves for use, a standard activation/calcination procedure was applied. Specifically, the sieves were placed in a furnace and subjected to a heating process at 350 °C under vacuum conditions for 3 h to remove any adsorbed water.

(2) Fabrication of ZSM-5@Celgard Separator

A slurry of ZSM-5 molecular sieves was prepared by mixing 80 wt% of activated ZSM-5 with 20 wt% polyvinylidene difluoride (PVDF) in N-methyl-2-pyrrolidone (NMP), provided by Sinopharm Group Chemical Reagent Co., Ltd. (Shanghai, China). This mixture was thoroughly blended to ensure uniform dispersion. The resulting slurry was then evenly coated onto the Celgard 2500 separator and dried at 80 °C for 6 h in a drying oven.

(3) Pressing Process of Prepared ZSM-5@Celgard Separator

To minimize the porosity between ZSM-5 particles within the ZSM-5@Celgard separator, an external pressure of 20 MPa was applied to press the coated separator. Following this pressing step, the ZSM-5 film was cut into small discs (Φ = 19 mm) and reactivated in a vacuum drying oven at 80 °C for 12 h.

### 2.2. Electrolytes and Electrode Preparation

1,2-Dimethoxyethane (DME) and lithium bis(fluorosulfonyl)imide (LiFSI) were procured from Aladdin Biochemical Technology Co., Ltd. (Shanghai, China), while acetylene black was sourced from Shenzhen Tianchenghe Technology Co., Ltd. (Shenzhen, China). Lithium iron phosphate (LFP) was purchased from Suzhou Duoduo Chemical Technology Co., Ltd. The 2 M LiFSI DME electrolyte was prepared inside an Ar-filled glove box (H_2_O < 0.01 ppm, O_2_ < 0.01 ppm). For electrode preparation, a cathode slurry was formulated by blending LFP powder, acetylene black, and PVDF in an 8:1:1 mass ratio using NMP as the solvent. The slurry was homogeneously coated onto carbon-coated aluminum (Al) foil current collectors using a doctor blade technique and dried at 80 °C for 12 h. The finished LFP electrodes were cut into circular plates (Φ = 13 mm). The area mass loading of the LFP electrode sheet was calculated after weighing to be 1.3 mg/cm^−2^.

### 2.3. Cell Assembly and Electrochemical Measurement

All batteries were assembled as 2032 coin-type cells within an argon-filled glove box (H_2_O < 0.01 ppm, O_2_ < 0.01 ppm). Li||Cu and Li||Li cells were tested at current densities of 0.5 mA cm^−2^ and 1 mA cm^−2^, respectively. LFP||Li cells operated within a voltage range of 2.5–4.0 V, with charge/discharge current densities calculated based on 1 C = 170 mAh/g (theoretical specific capacity of LiFePO_4_). The electrolyte volume was maintained at 60 μL for all coin cells.

### 2.4. Materials Characterizations

Scanning electron microscopy (SEM) was employed to examine the surface morphology of the lithium metal using a ZEISS Gemini SEM 500 instrument (Oberkochen, Germany). X-ray photoelectron spectroscopy (XPS) signals were collected from cycled Li metal anodes in Li||Li cells using a Thermo Fisher Scientific K-Alpha spectrometer (Waltham, MA, USA). Thermogravimetric analysis (TGA) was conducted on a TG 209 F1 Nevio instrument (NETZSCH-Gerätebau GmbH, Selb, Germany) over a temperature range of 30 °C to 500 °C in a nitrogen atmosphere. Raman spectra were acquired using a HORIBA LabRAM HR Evolution spectrometer (Kyoto, Japan).

### 2.5. Molecular Dynamics (MD) Simulations

The structures and dynamics of the electrolytes were investigated through molecular dynamics (MD) simulations. A simulation box containing 20 Li^+^ ions, 20 FSI^−^ anions, and 98 DME molecules was used to represent the 2 M LiFSI DME electrolyte. MD simulations were carried out using Materials Studio 2017 software. All molecules/ions were modeled using the Universal force field, with geometry optimization performed via Forcite Geometry Optimization. The simulations were run in an NVT ensemble for a duration of 500 picoseconds.

## 3. Results and Discussion

### 3.1. The Solvation Structures in ZSM Electrolytes

**Figure 2:**
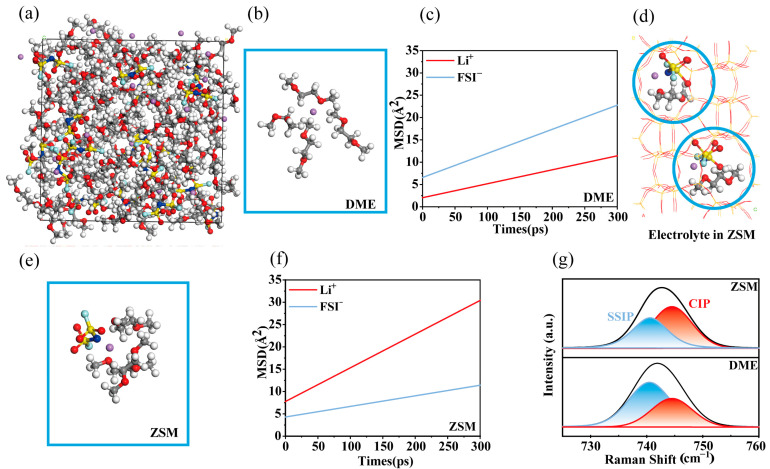
Solvation structures. (**a**) Snapshots of solvation structure in DME electrolytes calculated from MD simulations. (**b**) Typical solvation structures of DME electrolytes during the MD simulations. (**c**) The MSD of Li^+^ and FSI^−^ ions in DME electrolytes with the simulation time. (**d**) ZSM electrolytes where the DME electrolytes are incorporated into the ZSM-5 host. (**e**) Solvation structures of ZSM electrolytes during the MD operation. (**f**) The MSD of Li^+^ and FSI^−^ ions in ZSM electrolytes with the simulation time. (**g**) Raman spectra of DME and ZSM electrolytes.

Molecular dynamics (MD) simulations were conducted to investigate the structural and dynamic properties of the ZSM electrolytes. For comparison, 2 M LiFSI DME electrolytes (hereafter referred to as DME electrolytes) were also analyzed (Figure S1). In DME electrolytes (Figure 2a), Li^+^ ions were predominantly surrounded by DME molecules within their solvation shells, which is detrimental to the stability of the lithium metal interface (Figure 2b) [45]. Mean square displacement (MSD) simulations revealed that Li^+^ ions exhibited a lower diffusion rate compared to the relatively larger FSI^−^ anions (Figure 2c). This reduced diffusion efficiency can be attributed to the extensive coordination of Li^+^ with numerous DME molecules, hindering ion mobility. According to sand theory, such low Li^+^ diffusion efficiency can promote dendrite formation [46]. In contrast, when DME electrolytes passed through the ZSM-5-sieving channels (Figure 2d), a certain amount of FSI^−^ anions entered the solvation structures (Figure 2e), contributing to an anion-rich interphase. Furthermore, MSD simulations demonstrated an enhanced Li^+^ diffusion rate (Figure 2f), facilitating more uniform lithium metal deposition according to sand theory [46].

XRD tests on ZSM-5 molecular sieves show that their composition mainly consists of Al_2_O_3_ and SiO_2_ (Figure S2). The mass composition of the ZSM-5 molecular sieve powder was further determined by EDX analysis, in which the content of SiO_2_ was significantly higher (Figure S3). Raman spectroscopy was used for further probing the solvation structures in ZSM electrolytes (Figure 2g). Commercial ZSM-5 zeolite powders were fabricated into membranes (Figures S4–S12). ZSM ion-sieving membranes were impregnated with DME electrolytes for testing. Raman analysis showed that in DME electrolytes, the solvation structures were predominantly SSIPs at 741.1 cm^−1^, with few CIPs present at 743.9 cm^−1^. Conversely, in ZSM electrolytes, the S-N-S bending vibration in FSI^−^ shifted from an SSIP-dominant structure to a CIP-dominated one, indicative of anion-rich solvation structures forming on the lithium metal surface [47,48,49].

### 3.2. Electrochemical Performance of the Electrolytes

**Figure 3:**
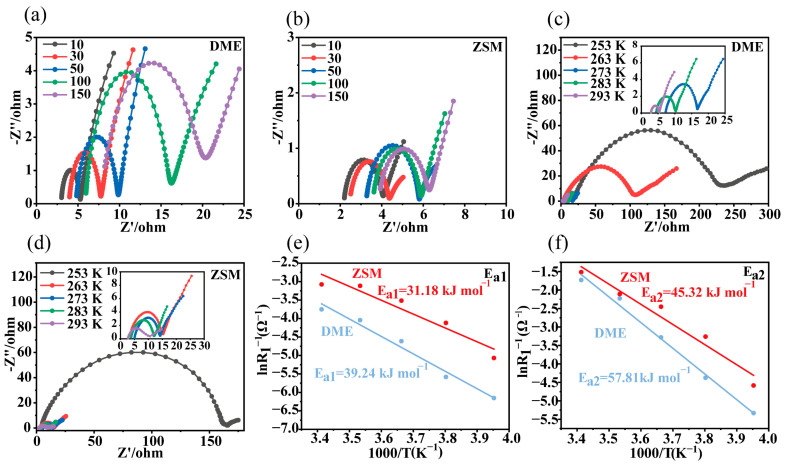
Electrochemical performance of DME and ZSM electrolytes. (**a**) Nyquist plots of the DME electrolyte at different cycles. (**b**) Nyquist plots of the ZSM electrolyte at different cycles. (**c**,**d**) Nyquist plots of Li||Li cells at various temperatures for both electrolytes. (**e**,**f**) Arrhenius behavior derived from the Nyquist plots of Li||Li symmetric cells, including a comparison of activation energies between R_1_ and R_2_.

Li||Li symmetric cells were assembled to perform electrochemical impedance spectroscopy (EIS) tests (Figure 3a,b and Table S1). For both electrolyte types, the ohmic impedance in the high-frequency region and the charge transfer impedance in the mid-frequency region increased over cycling. However, these impedance increases were significantly slower in ZSM cells, indicating improved interface stability. Moreover, the impedance values in ZSM cells were notably lower than those in DME cells, suggesting enhanced interfacial kinetics.

According to the equivalent circuit diagram (Figure 3c,d and Figure S13, and Table S2), E_a1_ represents the activation energy for Li^+^ diffusion in the SEI, while E_a2_ denotes the activation energy for stripping the solvation shell from lithium ions [45]. As shown in Figure 3e,f, E_a1_ in ZSM electrolytes was 31.18 kJ mol^−1^, lower than in DME electrolytes (39.24 kJ mol^−1^), indicating better Li^+^ diffusion efficiency in the SEI of ZSM electrolytes. Similarly, E_a2_ in ZSM electrolytes was 45.32 kJ mol^−1^, significantly lower than in DME electrolytes (57.81 kJ mol^−1^), implying faster Li^+^ transport kinetics at the lithium metal interface [47]. This result is significantly better than the data obtained in other studies based on LiFSI salts and DME solvent electrolytes (E_a1_ = 71.2 kJ mol^−1^, E_a2_ = 48.1 kJ mol^−1^) [45].

**Figure 4:**
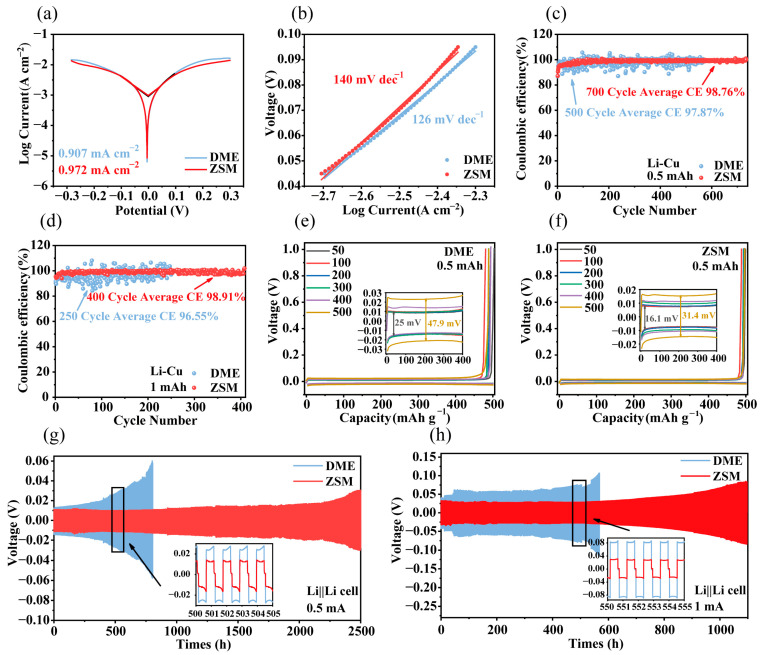
Electrochemical performance in DME and ZSM electrolytes. (**a**) Comparison of the Tafel curves observed in DME and ZSM electrolytes. (**b**) Comparison of Tafel slopes in DME and ZSM electrolytes. (**c**) Li||Cu cell performance over cycles at a current density of 0.5 mA cm^−2^. (**d**) Analysis of cycling performance for Li||Cu coin cells operating at a current density of 1 mA cm^−2^. (**e**) Polarization curves depicting the plating and stripping processes in DME electrolyte 0.5 mA cm^−2^. (**f**) Polarization curves depicting the plating and stripping processes in a ZSM electrolyte at 0.5 mA cm^−2^. (**g**) Voltage profiles of Li||Li cells at 0.5 mA cm^−2^. (**h**) Voltage profiles of Li||Li cells at 1 mA cm^−2^.

The Tafel test to calculate the exchange current density is an important method to evaluate the kinetics of electrode reactions, which can reflect the ease of charge transfer. By extending the polarization curves of the anode and cathode of the Tafel curve, the logarithm of the exchange current density can be obtained at the point of intersection. Tafel tests (Figure 4a) indicated a current density of 0.972 mA cm^−2^ in ZSM electrolytes, higher than in DME electrolytes (0.907 mA cm^−2^), signaling enhanced interfacial reaction kinetics in ZSM electrolytes, which is consistent with simulation results [48]. Additionally, the Tafel slope in ZSM electrolytes was markedly higher than in DME electrolytes (Figure 4b) [49].

CE tests were adopted for evaluating the long-term cycling stability of lithium metal anodes. At a current density of 0.5 mA cm^−2^ over 500 cycles (Figure 4c), DME electrolyte-based cells displayed an average CE of 97.87% with significant fluctuations. By contrast, ZSM electrolyte-based cells exhibited stable cycling for 750 cycles with an average CE of 98.76%, highlighting the superiority of ZSM-sieving channels for lithium metal interface stability. When the current density increased to 1 mA cm^−2^ (Figure 4d), DME cells sustained only 250 cycles with an average CE of 96.55%. In comparison, ZSM cells maintained an average CE of 98.91% for 400 cycles. Li||Cu cells based on ZSM electrolytes showed a polarization potential increase from 16.1 mV to 31.4 mV between the 50th and 500th cycles at 0.5 mA cm^−2^ (Figure 4e,f), demonstrating superior performance over DME electrolytes (from 25 mV to 47.9 mV). At 1 mA cm^−2^, ZSM cells still exhibited lower polarization potentials (Figures S14 and S15).

Li||Li symmetric cells were tested to investigate polarization potential during cycling in ZSM electrolytes. DME electrolyte-based cells experienced a sudden increase in polarization potential after 500 h at a testing current of 0.5 mA cm^−2^. Conversely, ZSM cells operated stably for 1000 h with minimal potential polarization increase (Figure 4g), confirming improved lithium metal interface stability, consistent with Li||Cu test results. At 1 mA cm^−2^, ZSM cells remained stable for 1000 h (Figure 4h).

### 3.3. Interfacial Properties of ZSM Electrolytes

**Figure 5:**
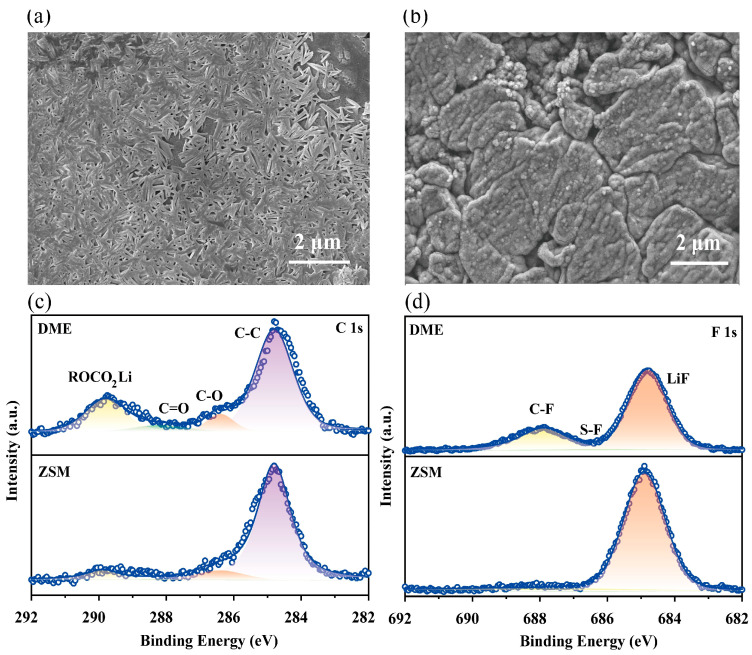
Structural evolution of lithium metal anodes. (**a**,**b**) SEM top-view image of the lithium metal anodes following 50 cycles using DME electrolytes and ZSM electrolytes. (**c**,**d**) XPS analysis of the lithium metal anodes post-cycling, showing C 1s (c), and F 1s (f) spectra for electrodes cycled in both DME and ZSM electrolytes.

Scanning electron microscopy (SEM) examined the lithium metal surface morphology after 50 cycles (Figure S16). DME electrolytes led to randomly distributed lithium dendrites on the lithium metal surface after continuous plating/stripping (Figure 5a), potentially causing short circuits. In stark contrast, ZSM cells exhibited a much smoother lithium metal surface with almost no visible dendrites (Figure 5b), demonstrating excellent interface stability.

X-ray photoelectron spectroscopy (XPS) was utilized to investigate the composition of the SEIs that formed on the lithium metal surface in ZSM electrolytes. Peaks at 289.8, 288.2, 286.4, and 284.8 eV in the C 1s spectrum (Figure 5c) corresponded to ROCO_2_Li, C=O, C-O, and C-C, respectively [50,51]. Compared to ZSM electrolytes, DME electrolytes showed more ROCO_2_Li composition in the SEIs, likely due to higher reactivity towards lithium metal, which could obstruct ion transport in the SEIs. In ZSM cells, an enhanced LiF characteristic peak appeared at 648.8 eV, which is beneficial for lithium metal interface stability (Figure 5d) [52,53,54].

### 3.4. Performance of LFP||Li Full Batteries

**Figure 6:**
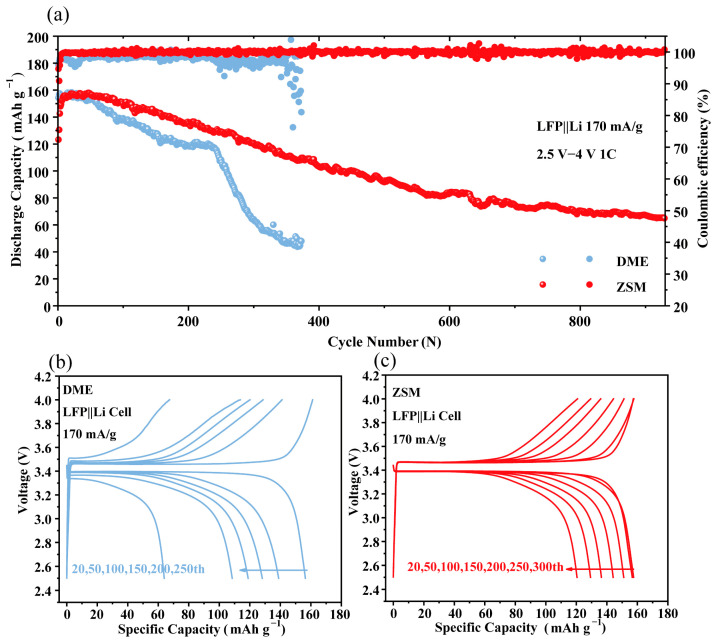
(**a**) Cycling performance and CE of LFP||Li cells cycled at 1 C between 2.5 and 4 V in different electrolytes. (**b**,**c**) The voltage profile corresponding to LFP||Li batteries with DME electrolytes and ZSM electrolytes.

To further demonstrate the effectiveness of ZSM electrolytes, LFP||Li batteries were tested at 1 C (Figure 6a). DME battery capacity sharply dropped after 200 cycles (capacity retention at 300 laps is only 41.33%). The results of this test are consistent with the study of Zhang et al. [55]. The DME electrolytes are unable to maintain a stable long cycling. However, ZSM batteries stably cycled 900 times with a capacity retention of 50.5%, showing improved stability. Voltage curves under different cycle times (Figure 6b,c) highlighted the superiority of ZSM batteries. After 200 cycles, the discharge capacity of DME batteries decreased to 109 mAh g^−1^, and this capacity rapidly decreased to 63 mAh g^−1^ after 250 cycles. In addition, the DME batteries showed a certain degree of voltage decay during charging and discharging, with the discharge voltage dropping from 3.4 V to 3.3 V. In contrast, the ZSM battery exhibited a discharge capacity of 121 mAh g^−1^ after 300 cycles without voltage degradation. These results underscore the critical role of the anion-rich interface in ZSM electrolytes for enhancing battery cycling stability. The inorganic-rich SEI film formed at the anion-rich interface has higher mechanical strength, induces uniform Li^+^ deposition, and avoids the growth of lithium dendrites. This is particularly important for the cycling performance of lithium metal batteries.

## 4. Conclusions

In this study, we utilized ZSM-5 zeolite molecular sieves as effective ion-sieving channels for the lithium metal anode interface without altering the bulk electrolyte structure. Li||Cu cells achieved an average CE of 98.76% over 700 h, and LiFePO_4_||Li batteries remained stable for 900 cycles at 1 C. The excellent electrochemical properties can be attributed to the following: (1) the uniform pore structure within the molecular sieve efficiently expels a portion of the solvent from the solvation structures, leading to an anion-rich interphase and promoting the formation of anion-derived SEIs; (2) sieved electrolytes have better interfacial dynamics and lower desolvation energy; and (3) the increased LiF component in these SEIs significantly enhances lithium metal interface stability. This design strategy may provide a new direction for effective interface regulation in lithium metal batteries, paving the way for advancements in high-energy-density storage systems.

## Data Availability

The original contributions presented in this study are included in the article/Supplementary Material. Further inquiries can be directed to the corresponding authors.

## Supplementary Materials

The following supporting information can be downloaded at: https://www.mdpi.com/article/10.3390/ma18112415/s1, Figure S1: Molecular structure of the LiFSI salt and DME solvent.; Figure S2: XRD diagram of ZSM-5 powder; Figure S3: (a) SEM image of ZSM-5 molecular sieve powder; (b-e) Elemental composition and EDX elemental Mapping of ZSM-5 molecular sieve powder; Figure S4: ZSM-5 molecular sieve powder; Figure S5: Molecular structure of ZSM-5 molecular sieve.; Figure S6: DSC profile of ZSM-5 Zeolite; Figure S7: SEM image of ZSM-5 molecular sieve powder; Figure S8: Optical picture of ZSM-5 membrane; Figure S9: **(**a) Optical picture of Celgard 2500 diaphragm; (b) Optical picture of ZSM-5 modified diaphragm; Figure S10: SEM image of ZSM-5 modified diaphragm surface; Figure S11: Cross-section observation of unpressed ZSM-5 modified diaphragm; Figure S12: Cross-section observation of pressed ZSM-5 modified diaphragm; Figure S13: The equivalent circuit models.; Figure S14: Polarization curves of plating/stripping process in DME electrolytes at 1 mA cm^-2^; Figure S15: Polarization curves of plating/stripping process in ZSM electrolytes at 1 mA cm^-2^; Figure S16: (a) Top-view SEM images of the Li-metal electrode with DME electrolytes after 50 cycles; (b) Top-view SEM images of the Li-metal electrode with ZSM electrolytes after 50 cycles; Table S1: The fitted parameters of Nyquist plots with different number of cycles; Table S2: The fitted parameters of Nyquist plots with different temperatures.
