# Supplementary material for: Anion-Enriched Interfacial Chemistry Enabled by Effective Ion Transport Channels for Stable Lithium Metal Batteries
Source: Materials (Basel). 2025 May 22;18(11):2415. doi: 10.3390/ma18112415 (PMC12156884; doi:10.3390/ma18112415)
Supplement: Supplementary file 1 [file materials-18-02415-s001.zip › materials-3640152-supplementary.pdf]

Supporting Information

Anion-Enriched Interfacial Chemistry Enabled by Effective Ion Transport Channels for Stable Lithium Metal Batteries

Yi Li[a], Hongwei Huang[a], Haojun Liu[a], Dedong Shan[a], Xuezhong He[b], Lingkai Kong[c], Jing Wang[c], Qian Li[a,*],Jian Yang[a,*]

[a] *College of Materials Science and Engineering, and Jiangsu Collaborative Innovation Center for Advanced Inorganic Function Composites, Nanjing Tech University, Nanjing, 211816, China*

[b] *Department of Chemical Engineering, and Guangdong Provincial Key Laboratory of Materials and Technologies for Energy Conversion, Guangdong Technion-Israel Institute of Technology, Shantou, 515063, China*

[c] *Longdu Laboratory for New Chemical Materials, Henan Province, Puyang, 457000, China*

[*]Corresponding author

E-mail address: liqian1004@njtech.edu.cn, yangjian1976@163.com.

The Supporting information includes the 16 Figures and 2 Tables.

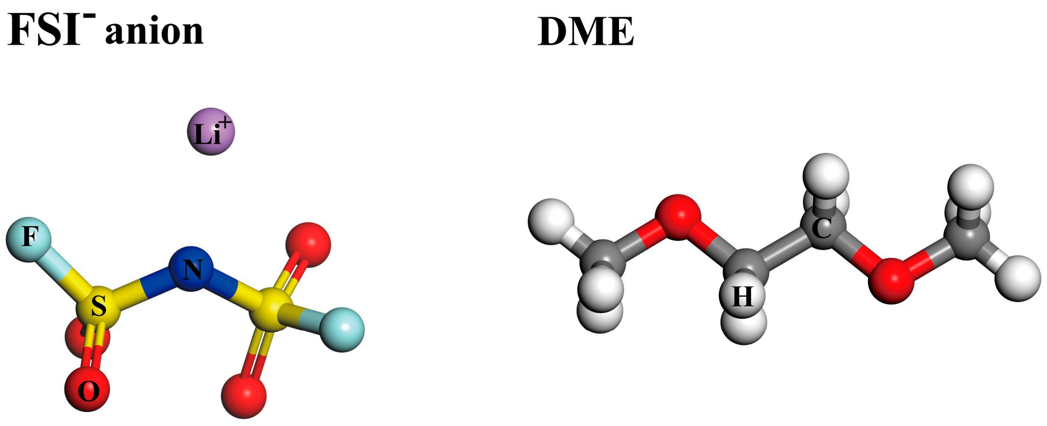

Figure S1. Molecular structure of the LiFSI salt and DME solvent.

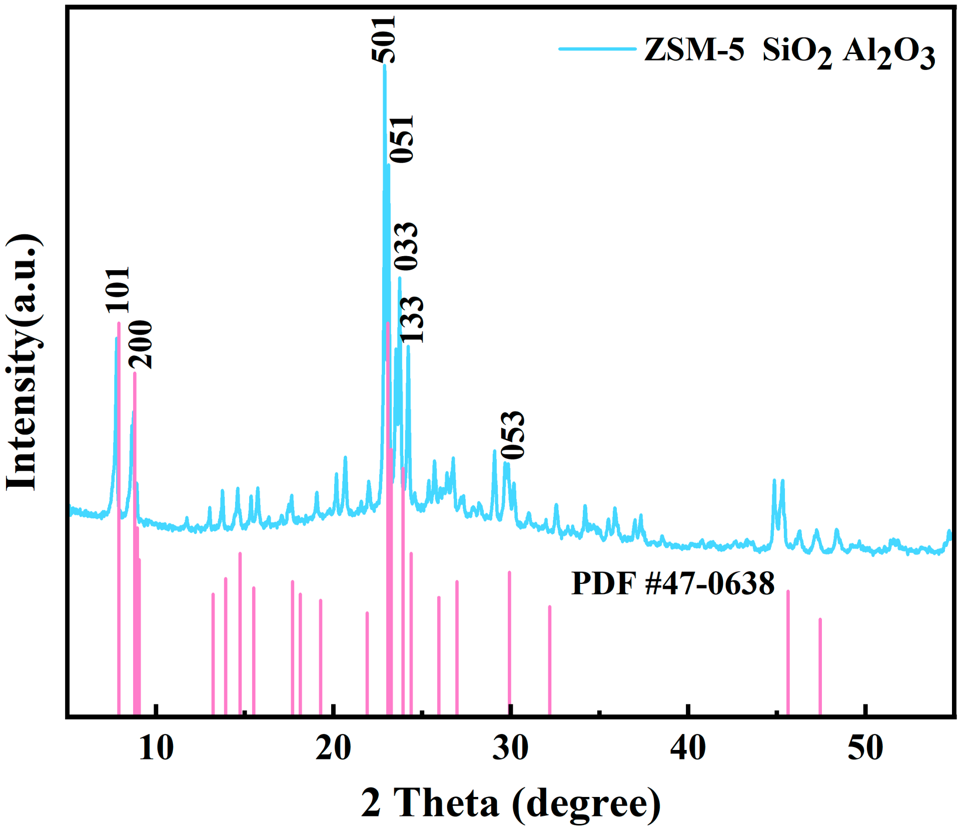

Figure S2. XRD diagram of ZSM-5 powder.

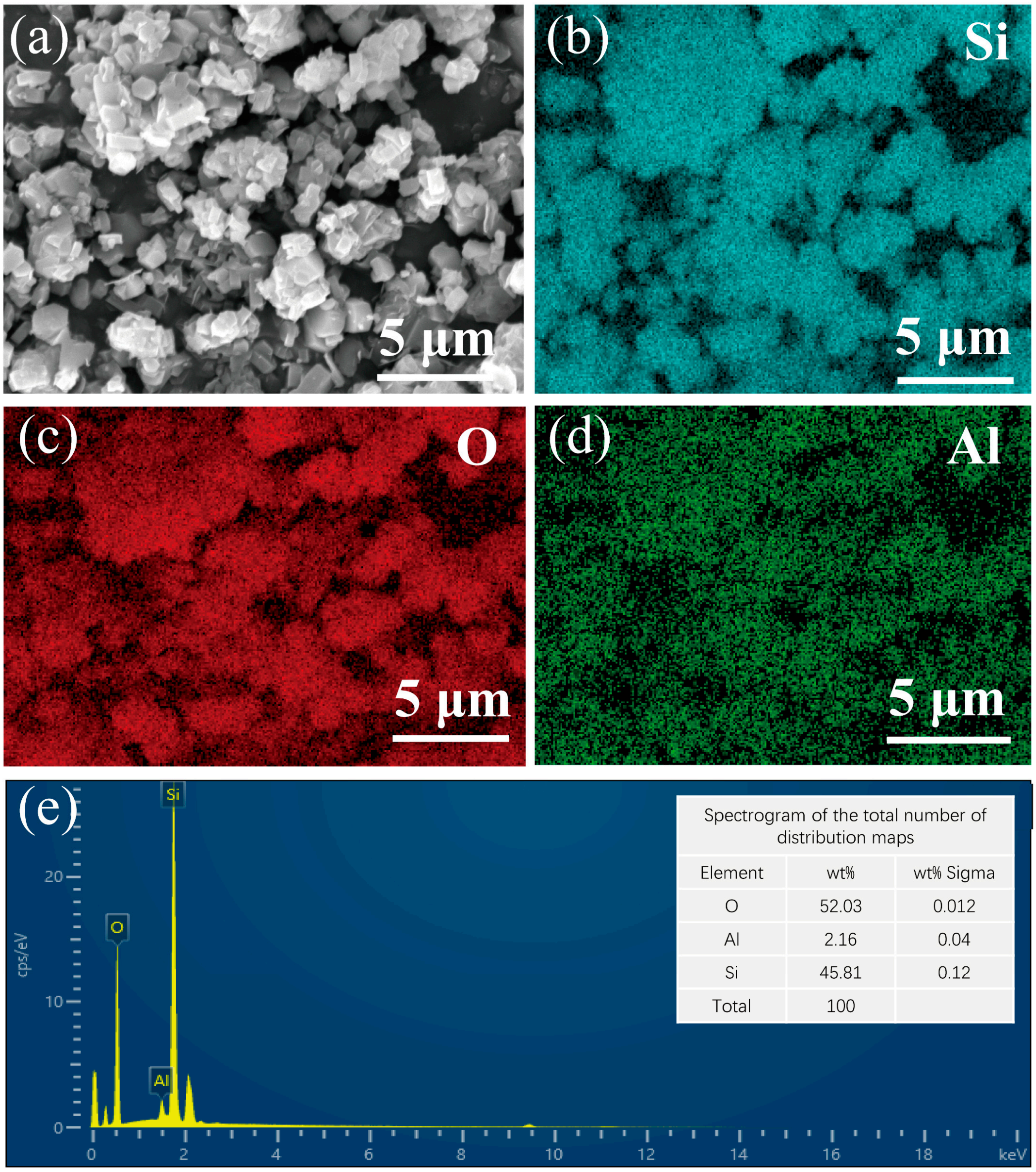

Figure S3. (a) SEM image of ZSM-5 molecular sieve powder; (b-e) elemental composition and EDX elemental mapping of ZSM-5 molecular sieve powder.

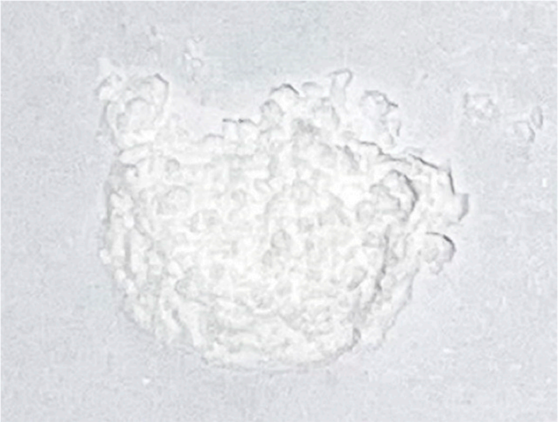

Figure S4. ZSM-5 molecular sieve powder.

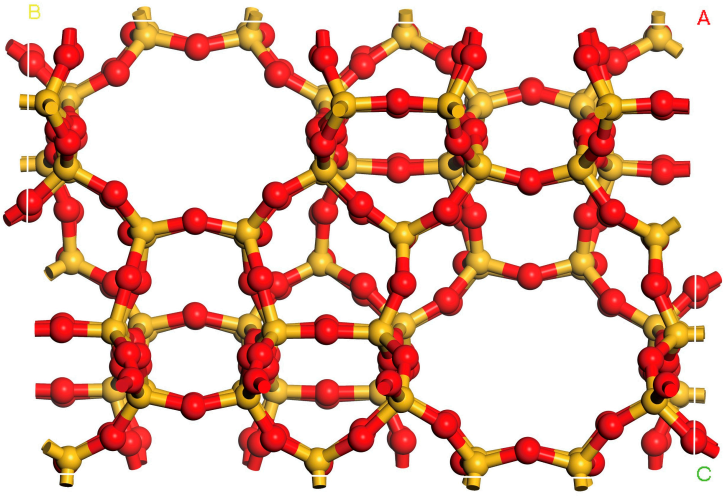

Figure S5. Molecular structure of ZSM-5 molecular sieve.

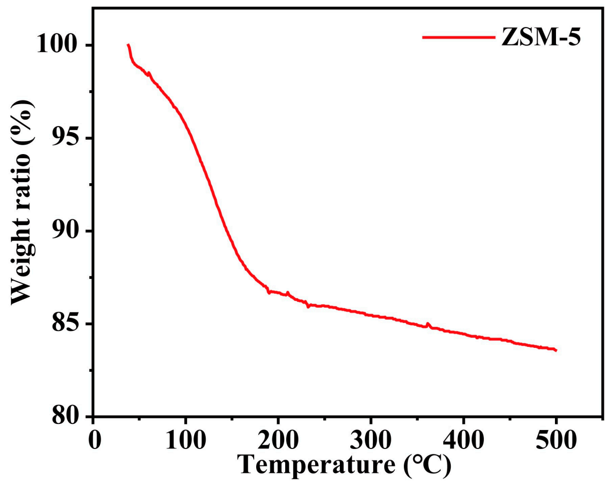

Figure S6. DSC profile of ZSM-5 zeolite.

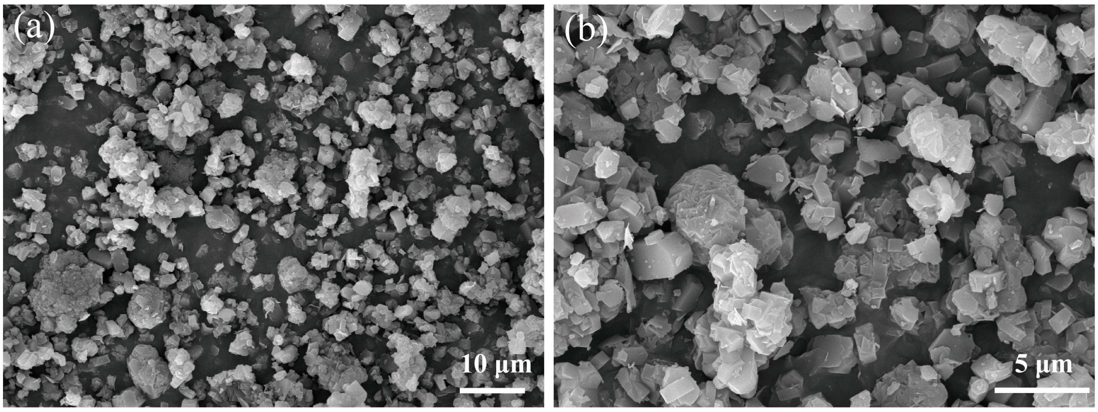

Figure S7. SEM image of ZSM-5 molecular sieve powder.

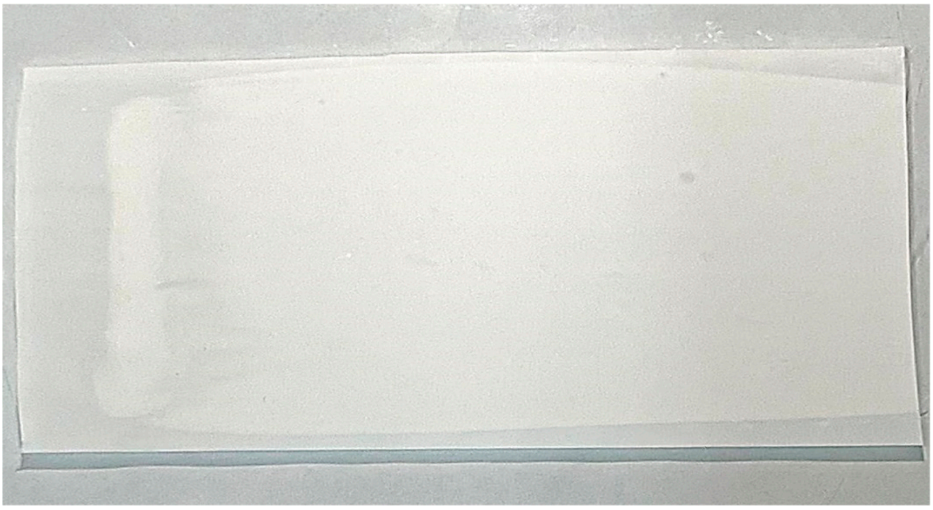

Figure S8. Optical picture of ZSM-5 membrane.

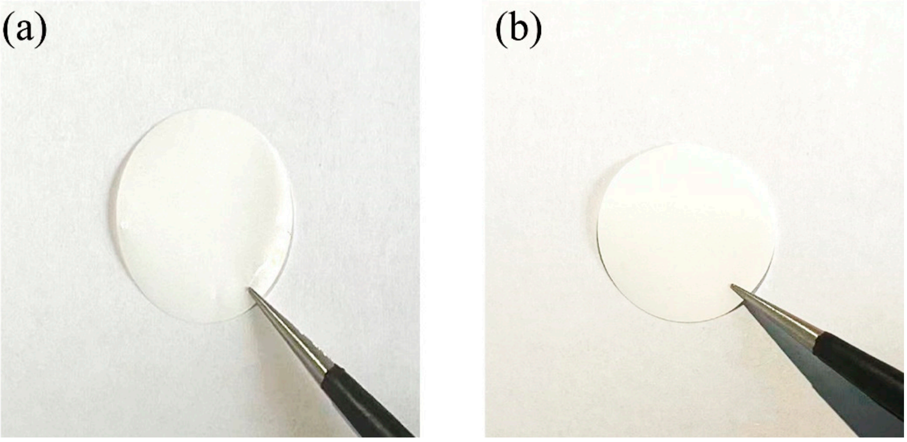

Figure S9. (a) Optical picture of Celgard 2500 diaphragm; (b) Optical picture of ZSM-5 modified diaphragm.

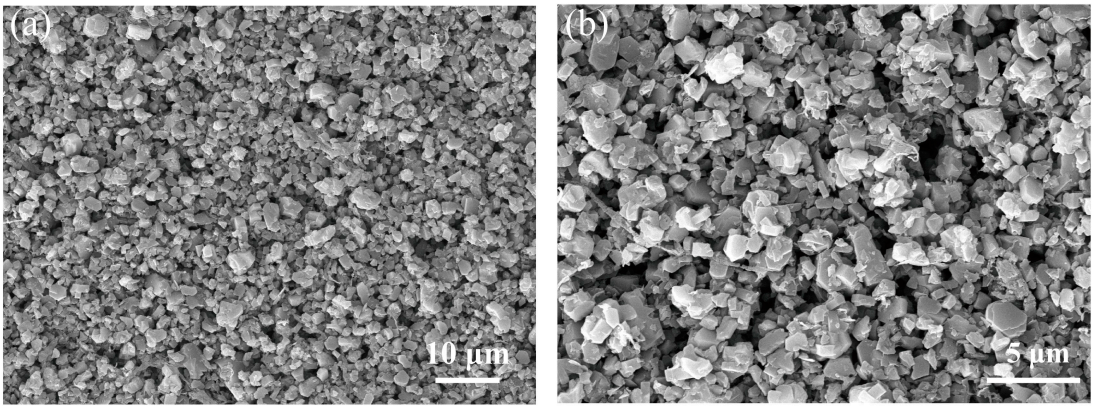

Figure S10. SEM image of ZSM-5 modified diaphragm surface.

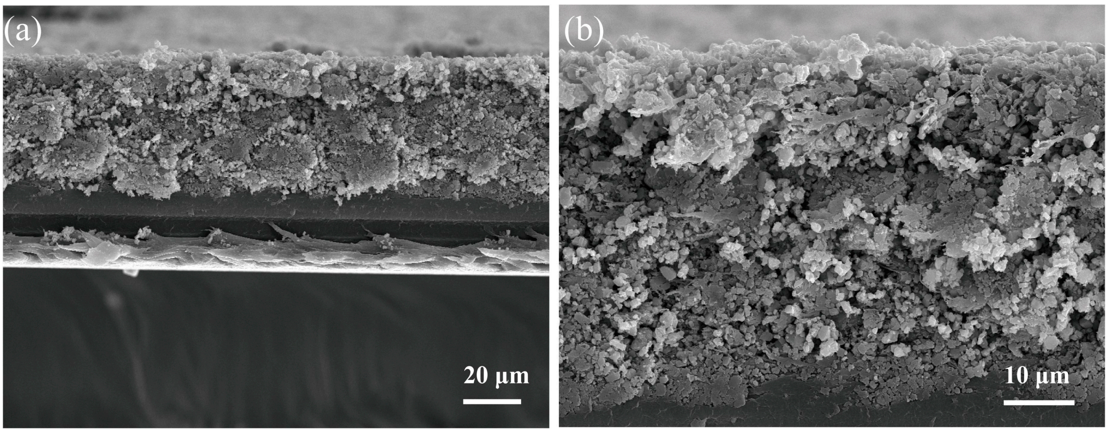

Figure S11. Cross-section observation of unpressed ZSM-5 modified diaphragm.

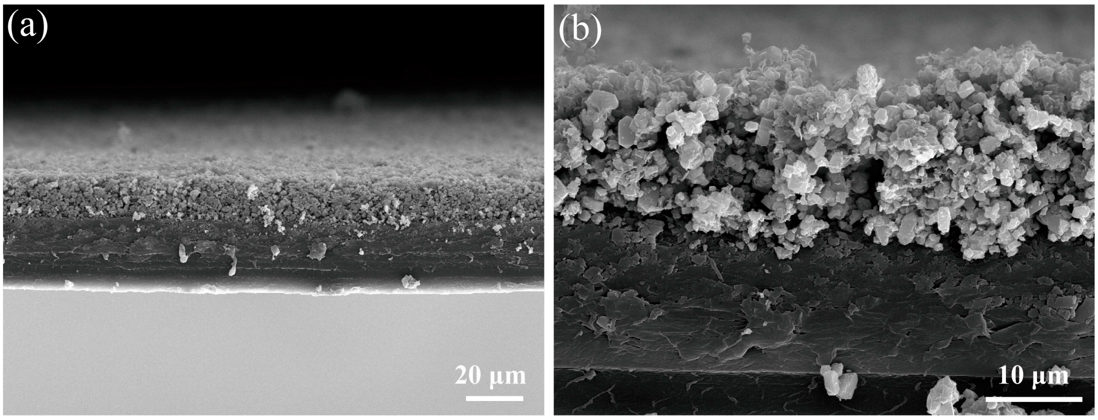

Figure S12. Cross-section observation of pressed ZSM-5 modified diaphragm.

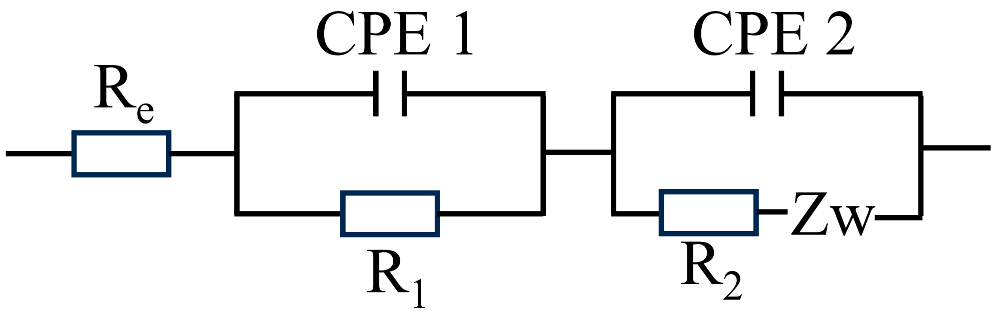

Figure S13. The equivalent circuit models. The R_e represents the resistance of electrolytes, and the R_1 arises from the impedance of the interfacial film on lithium anode, while the semi-circle of R_2 arises from the charge transfer process.

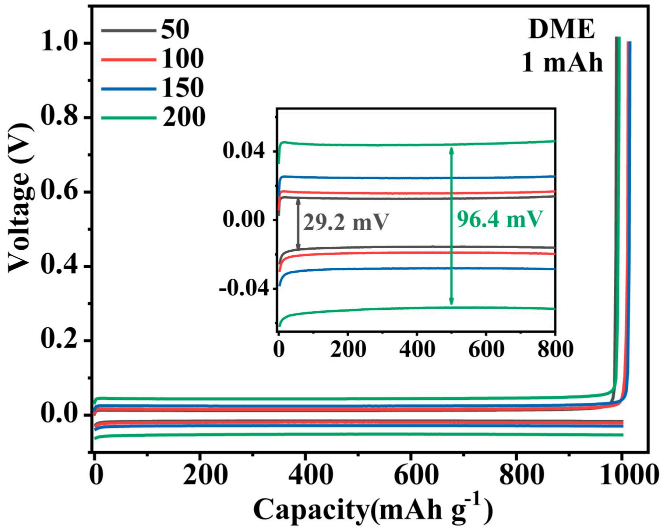

Figure S14. Polarization curves of plating/stripping process in DME electrolytes at 1 mA cm^{-2}.

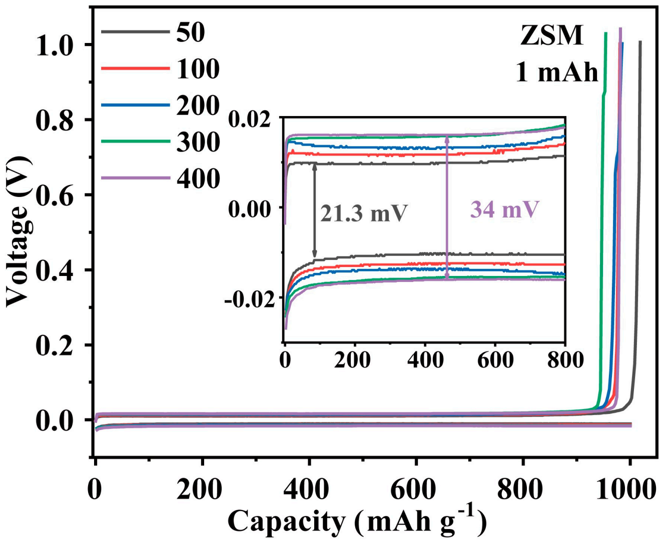

Figure S15. Polarization curves of plating/stripping process in ZSM electrolytes at 1 mA cm^{-2}.

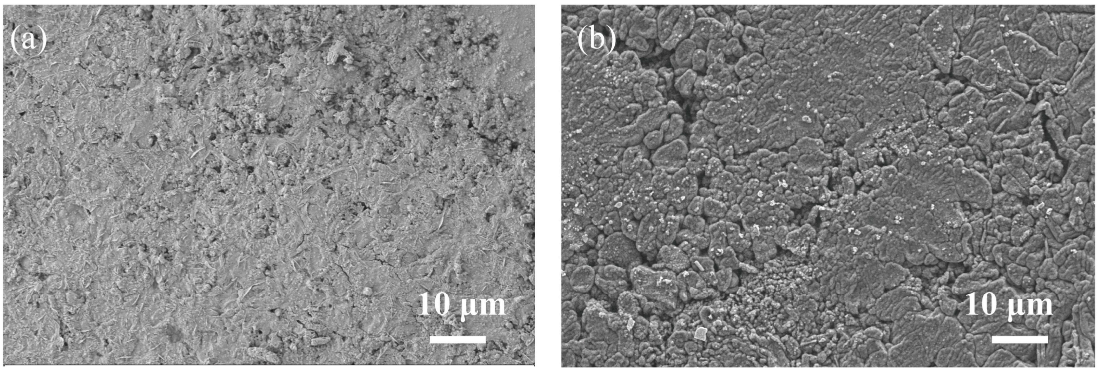

Figure S16. (a) Top-view SEM images of the Li-metal electrode with DME electrolytes after 50 cycles; (b) top-view SEM images of the Li-metal electrode with ZSM electrolytes after 50 cycles.

Table S1 The fitted parameters of Nyquist plots with different number of cycles.

	DME		ZSM	
	Re (Ohm)	R1 (Ohm)	Re (Ohm)	R1 (Ohm)
10th	2.982	3.265	2.179	2.839
30th	3.932	4.771	2.450	2.882
50th	4.766	6.021	3.246	3.047
100th	5.871	11.090	3.571	3.289
150th	7.618	13.098	3.841	3.388

Table S2 The fitted parameters of Nyquist plots with different temperatures.

	DME		ZSM	
	Re (Ohm)	R1 (Ohm)	Re (Ohm)	R1 (Ohm)
253 K	18.290	206.731	4.846	97.956
263 K	10.467	79.614	4.135	26.173
273 K	7.413	26.597	3.834	11.632
283 K	4.368	8.272	3.332	6.244
293 K	3.496	5.634	2.864	4.551
